# TCAD Simulation Study of Electrical Performance of a Novel High-Purity Germanium Drift Detector

**DOI:** 10.3390/mi16020229

**Published:** 2025-02-17

**Authors:** Mingyang Wang, Zheng Li, Bo Xiong, Yongguang Xiao

**Affiliations:** 1 School of Materials Science and Engineering, Xiangtan University, Xiangtan 411105, China; 202131550147@smail.xtu.edu.cn (M.W.); boxiong65190@163.com (B.X.); 2 College of Integrated Circuits, Ludong University, Yantai 264025, China

**Keywords:** high-purity germanium drift detector, TCAD simulation of electrical performance, heavy ion incidence

## Abstract

High-purity germanium (HPGe) detectors occupy a prominent position in fields such as radiation detection and aerospace because of their excellent energy resolution and wide detection range. To achieve a broader detection range, conventional HPGe detectors often need to be expanded to cubic-centimeter-scale volumes. However, this increase in volume leads to a large detector area, which in turn increases the detector capacitance, affecting the detector’s noise level and performance. To address this issue, this study proposes a novel high-purity germanium drift detector (HPGeDD). The design features a small-area central collecting cathode surrounded by concentric anode rings, with a resistive chain interposed between the anode rings to achieve self-dividing voltage. This design ensures that the detector’s capacitance is only related to the area of the central collecting cathode, independent of the overall active area, thus achieving a balance between a small capacitance and large active area. Electrical performance simulations of the novel detector were conducted using the semiconductor simulation software Sentaurus TCAD (P-2019.03). The results show a smooth electric potential distribution within the detector, forming a lateral electric field, as well as a lateral hole drift channel precisely directed toward the collecting cathode. Furthermore, simulations of heavy ion incidence were performed to investigate the detector’s carrier collection characteristics. The simulation results demonstrate that the HPGeDD exhibits advantages such as fast signal response and short collection time. The design proposal presented in this study offers a new solution to the problem of excessive capacitance in conventional HPGe detectors, expands their application scope, and provides theoretical guidance for subsequent improvements, optimizations, and practical manufacturing.

## 1. Introduction

Since the mid-1970s, with the continuous improvement in material purification technologies, particularly the rapid development of zone melting techniques, the impurity concentration in germanium crystals has been significantly reduced [1]. As a type of semiconductor detector, HPGe detectors demonstrate distinct advantages over silicon-based detectors. This superiority primarily stems from the fact that germanium possesses a higher atomic number, a lower average ionization energy, and a higher charge carrier mobility. These physical characteristics enable HPGe detectors to generate larger signals when detecting the same amount of radiation energy. Consequently, compared to silicon-based detectors, HPGe detectors theoretically exhibit wider energy responses, faster signal response times, higher collection rates, and stronger signal outputs. Additionally, HPGe detectors have the capability to be stored at room temperature and perform optimally when operated in a liquid-nitrogen-cooled environment [2,3].

HPGe detectors exhibit practical application value in the detection of heavy ions and other radiation particles [4,5]. Due to their extremely low internal radioactivity levels and high energy resolution, HPGe detectors have become key devices in the field of dark matter detection. When external radiation particles enter the sensitive volume of the detector, they induce the generation of electron–hole pairs. Under the influence of the internal electric field, these charge carriers are efficiently collected by the collecting electrodes, converting the radiation signal into an electrical signal. For example, the CDEX research team, composed of Chinese scientists, utilizes a ton-scale point-contact electrode HPGe detector array system to conduct direct dark matter detection experiments at the China Jinping Underground Laboratory [6]. The aim is to detect light dark matter particles with masses less than 10 GeV/c^2^, obtaining information such as the energy and position of the incident particles.

On the other hand, HPGe detectors hold potential application value in the field of X-ray pulsar navigation. Pulsars, as the most precise natural astronomical clocks, exhibit extremely stable rotation periods, with typical values ranging from milliseconds to seconds with a time precision better than 10^−7^ s [7,8,9,10,11]. These stable rotation periods make pulsars ideal candidates for various applications, including X-ray pulsar navigation, which is a novel autonomous navigation technology for spacecraft, utilizing pulsars as navigation beacons to provide autonomous navigation services for spacecraft in near-Earth orbits, deep space, and interstellar space [12,13,14,15,16,17]. Semiconductor detectors are the core components of X-ray pulsar navigation systems. When X-rays enter the depletion region of a semiconductor detector, they interact with material atoms to generate electron–hole pairs, which are then absorbed by the collecting electrodes under the influence of an electric field, thereby converting the X-ray signals emitted by pulsars into electrical signals.

Currently, silicon-based detectors have been applied in X-ray pulsar navigation systems [18]. Due to their superior material properties compared to silicon-based detectors, HPGe detectors offer performance advantages, such as further extension of the detection range and improved energy resolution, making them promising candidates for future development. With the further advancement of space cooling technologies, an aerospace-grade pulse tube cooler, successfully developed by a research team at the Institute of Physics and Chemistry of the Chinese Academy of Sciences, has been applied in the aerospace field, providing a reliable low-temperature liquid nitrogen environment. This has made it possible to realize the application of HPGe detectors in the field of X-ray pulsar navigation.

The application of HPGe detectors in the fields of radiation particles and X-ray detection is fundamentally based on the photoelectric effect of germanium crystals. When an HPGe detector is subjected to external radiation, the germanium crystal within the depletion region undergoes ionization, generating electron–hole pairs. Under the influence of the internal electric field, these charge carriers drift toward the corresponding electrodes and are ultimately collected by the detector’s electrodes. The resulting electrical signals are then amplified and processed for analyzing the properties of the radiation energy.

This has greatly promoted the remarkable advancement in HPGe detectors. HPGe detectors are primarily classified into two types: planar and coaxial. Planar detectors, distinguished by their compact structure, are particularly suitable for applications in confined spaces or specific scenarios, and they demonstrate excellent performance in measuring medium-energy to high-energy charged particle, nuclear radiation, as well as low-energy gamma rays [19]. Coaxial detectors, on the other hand, leverage their advantages of long axial detection length and large sensitive volume, making them an ideal choice for measuring high-energy rays with strong penetration capabilities [20].

HPGe detectors typically feature an ultra-large sensitive volume, aimed at expanding the detection range, capturing more detection signals, enhancing the signal collection capability, improving the detection efficiency, and, thus, more accurately reflecting the characteristics of the object being measured [21]. However, the increased thickness of the large-sized sensitive volume also leads to an increase in capacitance, which adversely affects the detector’s noise level and overall performance. Attempting to reduce capacitance by decreasing size often comes at the expense of the detector’s active area for detection. Therefore, we are faced with the challenge of effectively reducing capacitance while ensuring that the detector’s active area is not compromised.

To address the challenge of expanding the detection area of HPGe detectors while maintaining low capacitance, this study proposes an innovative detector design. The HPGe detector is designed as a large-area drift detector structure with a small-area central collecting cathode surrounded by 20 concentric anode rings, with a resistor chain capable of automatic voltage division added between the anode rings. Based on the structural characteristics of semiconductor drift detectors [22], this design ensures that the output capacitance of the detector is solely dependent on the area of the central collecting cathode, rather than the entire active area. Therefore, this design effectively increases the detector’s detection area while maintaining low capacitance, thereby reducing series noise and enhancing the overall performance of the detector.

Meanwhile, the design and optimization process of the novel HPGeDD proposed in this paper, which has not yet been actually manufactured, is fraught with challenges and uncertainties. Due to the high cost of HPGe wafers and the large number required for preliminary experiments, blindly proceeding with actual manufacturing without adequate theoretical groundwork would result in enormous resource wastage. Therefore, conducting preliminary TCAD simulation and modeling of the detector electrical performance is particularly important.

For this purpose, the semiconductor simulation software Sentaurus TCAD was utilized in this paper to conduct electrical performance simulations of the HPGeDD. The simulation results indicate that the electric potential distribution within the detector is smooth, and the transverse electric field parallel to the electrode direction effectively facilitates the collection of induced holes, forming a hole drift channel that precisely directs toward the collecting cathode within the sensitive region. Furthermore, simulations of heavy ion incidence on the HPGeDD under operating conditions were carried out. The process of electric signal generation in the detector is observed. It has also been verified that the HPGeDD exhibits characteristics such as fast response time and short collection time. The simulation results presented in this paper not only aid in a better understanding of the structural features and working principles of the HPGeDD but also provide theoretical guidance for the future actual manufacture of HPGeDDs.

## 2. Detector Structure

**Figure 1:**
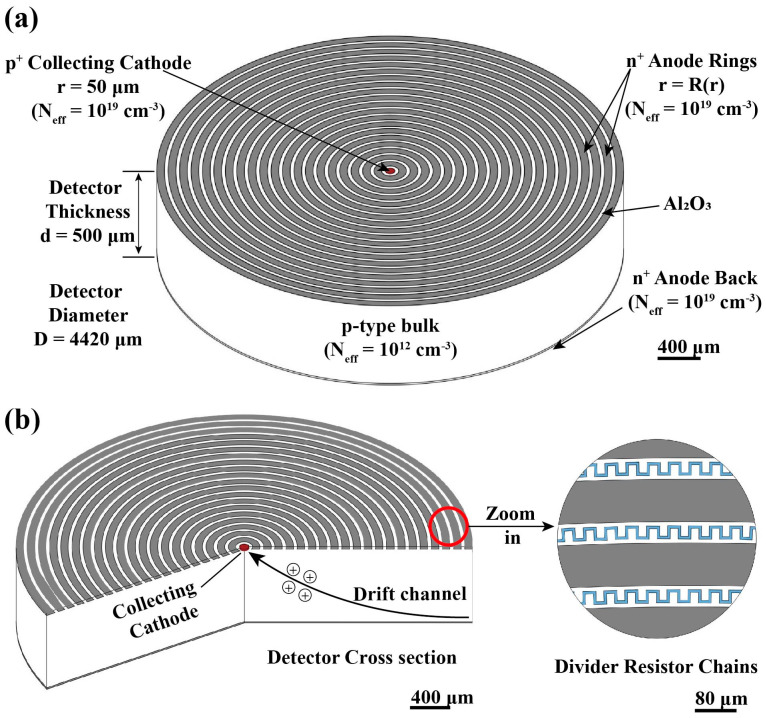
Schematic diagram of the HPGeDD. (**a**) Structure of the concentric anode rings HPGeDD. (**b**) Cross-sectional view of the HPGeDD and a locally enlarged view of the resistor chain.

As shown in Figure 1a, the HPGeDD is designed with a concentric ring structure. The detector substrate is made of p-type, high-purity germanium with a resistivity of 10,000 Ω·cm and a light doping concentration of 1 × 10^12^/cm^3^, with an overall thickness of 500 μm. As illustrated in Figure 1b, a central collecting cathode with a radius of 50 μm and a thickness of 1 μm is located at the very center of the detector. This cathode is fabricated from p-type, high-purity germanium with a heavy doping (ion implantation, etc.) concentration of 1 × 10^19^/cm^3^ and is primarily responsible for collecting the induced hole signals. Surrounding the central cathode are 20 concentric anode rings, arranged with an equal spacing of 30 μm. Each anode ring has a width of 80 μm and a thickness of 1 μm. The anode rings are made of n-type, high-purity germanium with a heavy doping (ion implantation, etc.) concentration of 1 × 10^19^/cm^3^.

Between adjacent anode rings, resistor chains are formed through the ion implantation of heavily doped n-type, high-purity germanium with a concentration of 1 × 10^19^/cm^3^ and a thickness of 1 µm is placed. Additionally, the electrode contact layer is made of aluminum, and Al_2_O_3_ is used as the passivation layer between the electrodes to ensure effective isolation between the electrodes.

## 3. Design of Voltage-Dividing Resistor Chains

**Figure 2:**
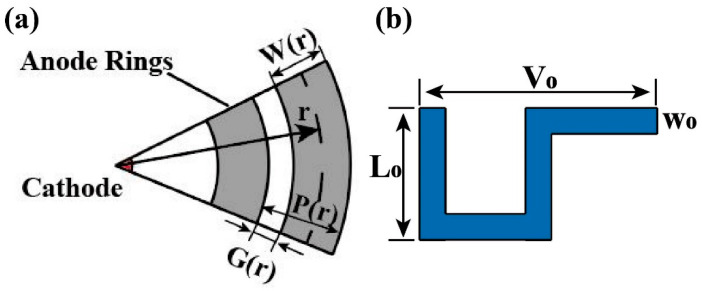
Design diagram of the voltage divider resistor chain for HPGeDD: (**a**) schematic diagram of the concentric anode rings for HPGeDD; (**b**) single resistor chain segment of the HPGeDD.

As shown in Figure 2a, to achieve the autonomous voltage division function of the HPGeDD, we incorporated resistor chains between the anode rings. Figure 2b presents a schematic of a single segment in the voltage-dividing resistor chain, which is constructed by connecting multiple such segments in a series. Each resistor chain connects to two adjacent anode rings at its two ends. Since an equal increment in the voltage division is required between each anode ring, we need to add 19 resistor chains of equal length between the first anode ring and the 20th anode ring based on the actual requirements and determine the corresponding arc degrees for the resistor chains configured between the anode rings.

As shown in Figure 2, $r_{1}$ is the radial distance between the cathode center and the first anode ring, r is the radial distance between the cathode center and the r-th anode ring, r = R is the radial distance between the cathode center and the outermost 20th anode ring, *G*(*r*) is the gap spacing between adjacent anode rings, *W*(*r*) is the width of the anode ring, and *P*(*r*) is the pitch at r between adjacent anode rings. We assumed the following in our simulation:

(1) $$W\left(r\right)=W_{0}=constant=80\;\mu m$$

(2) $$G\left(r\right)=G_{0}=constant=30\;\mu m$$

(3) $$P\left(r\right)=W\left(r\right)+G\left(r\right)=110\;\mu m$$

The first ring has an $r=r_{1}=120\;\mu m$, and the last ring has an r=R=2.21 mm. The resistive divider chain is located between adjacent anode rings with n^+^ implantation. The width of each resistor chain segment is $w_{0}$, the length is $L_{0}$, and the length of one unit is $V_{0}$. Within the interval of an anode ring with a radius of $r+\frac{1}{2}{(G}_{0}+W_{0})$, the arc length of the resistor chain corresponds to a radian measure of θ(r). k(r) represents the number of segments corresponding to each resistor chain.

The total length of the resistive chain is as follows:

(4) $$L\left(r\right)=k(r)\cdot (2L_{0}+V_{0})$$

(5) $$\theta (r)\cdot [r+\frac{1}{2}{(G}_{0}+W_{0})]=k(r)\cdot V_{0}$$

The resistance between the r-th ring and the $r+P_{0}$-th ring is as follows:

(6) $$\Omega (r)=\frac{\rho_{s}\cdot L(r)}{w_{0}}$$

or

(7) $$\Omega (r)=\frac{\rho_{s}\cdot k(r)\cdot (2l_{0}+V_{0})}{w_{0}}$$

The voltage between the r-th ring and the $r+P_{0}$-th ring is as follows:

(8) $$\Delta V\left(r\right)=I\cdot \Omega \left(r\right)=\frac{I\cdot \rho_{s}\cdot k(r)\cdot (2l_{0}+V_{0})}{w_{0}}$$

The following is the total number of anode rings (N):

(9) $$N=\frac{\left(R-r_{1}\right)}{P_{0}}+1$$

The outermost ring voltage is as follows:

(10) $$V_{out}=232\;V\left(r=R\right)$$

The innermost ring voltage is as follows:

(11) $$V_{E1}=80\;V\left(r=r_{1}\right)$$

(12) $$\Delta V\left(r\right)=constant=\frac{\left(V_{out}-V_{E1}\right)}{\left(\frac{R-r_{1}}{P_{0}}\right)}=8\;V$$

Appropriate values for $P_{0},\;G_{0}+W_{0}$, I, and $V_{out}-V_{E1}$ need to be selected to determine k(r) and $\theta \left(r\right)$, as follows:

(13) $$P_{0}=G_{0}+W_{0}=0.011\;cm$$

(14) $$I=3\times 10^{-7}\;A$$

(15) $$V_{out}-V_{E1}=152\;V$$

(16) $$\rho_{s}=2000\;\Omega$$

(17) R=2210 μm=0.221 cm

Let $L_{0}=25\;\mu m$, $V_{0}=50\;\mu m$, and $w_{0}=5\;\mu m$. Because $\theta \left(r_{1}\right)=2\pi$, substituting $r=r_{1}$ into Equation (5) leads to the following:

(18) $$k\left(r_{1}\right)=22$$

Assume the following:

(19) $$k\left(r\right)=k\left(r_{1}\right)$$

That is, the number of segments corresponding to each resistor chain is 22. From Equation (5), we know the following:

(20) $$\theta \left(r\right)=2\pi \cdot \frac{k(r)\cdot V_{0}}{r+\frac{1}{2}(G_{0}+W_{0})}$$

The results of substituting the corresponding values into Equation (20) yields are provided in Table 1.

By adding the corresponding arc length of the resistor chain in the appropriate positions based on the aforementioned radian values, $\theta \left(r\right)$, a voltage division effect can be achieved.

## 4. Simulation Results of Detector Electrical Performance

This study employs the semiconductor simulation software Sentaurus TCAD (P-2019.03) to conduct an in-depth investigation into the electrical performance of the HPGeDD. We utilize carrier transport equations, the SRH (Shockley–Read–Hall) recombination model, and the density of states model to analyze this device. For material parameters, we directly adopt those built into the Sentaurus TCAD software. In the mobility model, we consider carrier scattering by ionized impurities and incorporate the carrier mobility degradation model (DopingDep), the carrier velocity saturation model in high electric fields (HighFieldSat), and the mobility degradation model due to surface roughness scattering (Enormal). Regarding the density of states model, taking into account the doping effects during the simulation process, we employ the effective intrinsic density model (OldSlotboom). In the recombination model, the corresponding carrier generation and recombination processes are implemented through the carrier continuity equation, with the SRH recombination mechanism being selected. Additionally, an interface charge density of −4 × 10^11^ q/cm^2^ is set between Al_2_O_3_ and Ge.

To further enhance the accuracy of our simulations, we carefully defined the mesh size for the entire device. Specifically, the maximum mesh setting for the x/y/z coordinates is 8 μm, while the minimum mesh setting is 3 μm. Moreover, we refined the mesh at material interfaces and locations where the doping concentration changes. This refinement starts with an initial mesh size of 2 μm and increments it by a factor of 1.5 to ensure a more precise simulation of these critical areas.

**Figure 3:**
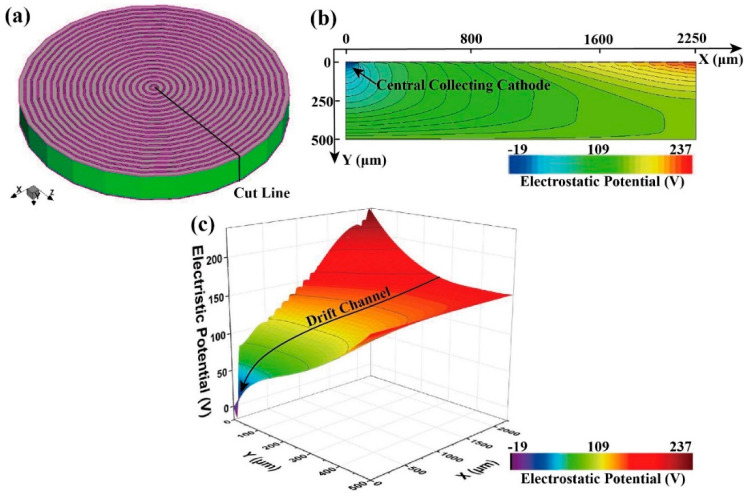
Electrical simulation images of the HPGeDD: (**a**) 3D simulation image of the HPGeDD, with a two-dimensional cross-section taken at a tangential position; (**b**) potential distribution map of the two-dimensional cross-section; (**c**) 3D potential distribution map of the HPGeDD. The drift channel is marked with a black line with an arrow.

As shown in Figure 3a, a three-dimensional (3D) image of the HPGeDD is displayed. Since the model adopted in this study is a concentric ring detector with a highly symmetrical structure, we selected a representative tangential position at half of the detector’s cross-section for investigation. In the simulation experiment, a voltage of 0 V is applied to the central cathode, 80 V to the first anode ring, 232 V to the last anode ring, and 152.5 V to the bottom anode plate.

Figure 3b presents the electric potential distribution of the HPGeDD. In the upper half of the detector, the potential is lowest in the collection cathode area located at the center on the left side, and it gradually increases from left to right. In the lower half, the potential of the bottom anode plate remains constant. By further observing Figure 3c, which shows the 3D potential distribution across a cross-section of the detector, we can see that a low-potential valley forming a carrier drift channel within the sensitive area of the detector. This channel extends smoothly from the edge of the detector to the central collection cathode (as indicated by the black arrow in the figure), providing favorable conditions for holes within the substrate to move toward and be absorbed by the central cathode.

When induced holes are generated inside the detector, they preferentially drift into this low-potential hole drift channel. Subsequently, the holes move gradually toward the central collection cathode along the direction of decreasing potential within the drift channel and are ultimately collected. Additionally, the potential changes continuously and smoothly around the anode rings, with no abrupt potential variations. This allows for the holes excited by incident particles to drift accurately into the drift channel and be collected. These results indicate that the design presented in this paper has achieved the expected goals.

**Figure 4:**
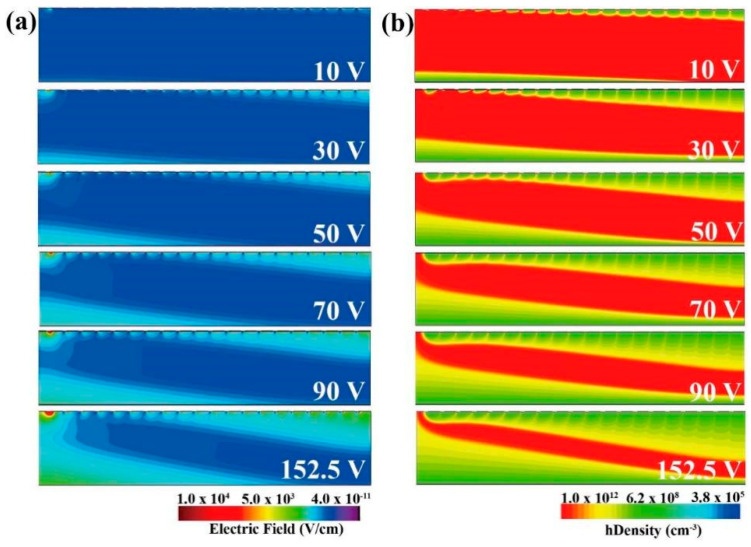
Distributions of the internal electric field and hole density in the HPGeDD as a function of voltage, with bottom anode plate biases from 10 V to 152.5 V: (**a**) electric field; (**b**) hole density.

To further explore the depletion process within the HPGeDD, as well as the formation mechanism of the electric field and the hole drift channel, we recorded the distributions of electric field and hole concentration of the detector under different voltages. Figure 4a presents the electric field distribution within the detector under various biasing conditions. As the voltage gradually increases, the electric field strength in the upper half of the detector enhances, gradually pushing the low-field regions toward the vicinity of the drift channel. Simultaneously, the electric field in the lower half of the detector also intensifies with increasing voltage, similarly squeezing the low-field regions toward the central drift channel. When the detector reaches full depletion, the smooth electric field within its sensitive area effectively promotes the separation and drift of electron–hole pairs generated by radiation ionization, thereby generating an electrical signal. The induced holes will move toward the drift channel under the action of the electric field. Inside the channel, there is a gradually increasing electric field pointing toward the collection cathode, and the induced holes continue to drift to the central collection cathode under the influence of this electric field.

The distributions of the detector’s hole concentrations under different biasing voltage conditions are presented in Figure 4b. As the biasing voltage gradually increases, the hole depletion zones extend from both surfaces toward the middle of the detector, gradually forming a hole drift channel, most visible at a bias of 152.5 V. It is noteworthy that the path for holes in the carrier drift channel to transport to the central collection cathode is the shortest. This short drift path effectively reduces the diffusion and recombination losses of holes during the drift process, thereby improving the detector’s collection efficiency and energy resolution.

**Figure 5:**
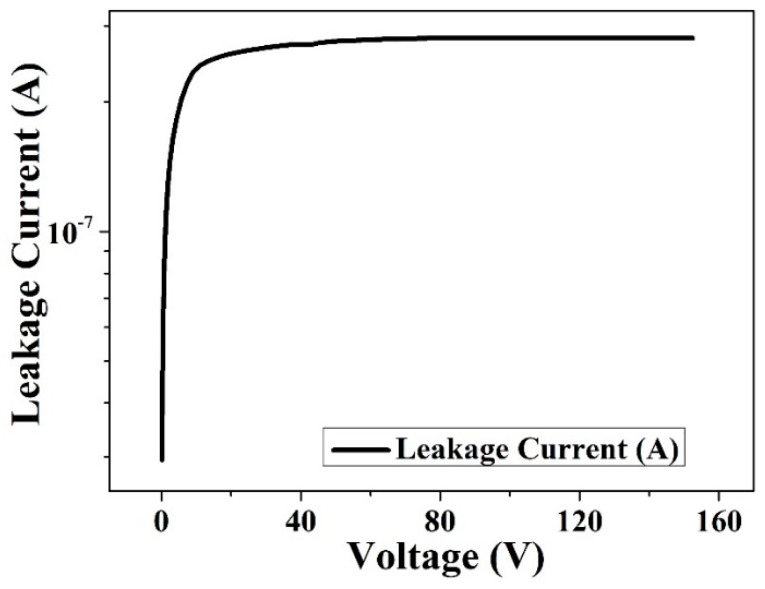
Leakage current versus voltage curve for an HPGeDD.

As shown in Figure 5, the curve of the leakage current versus voltage for the HPGeDD is presented. The leakage current of the detector primarily originates from impurities and lattice defects in the material. These impurities and defects give rise to the generation–recombination current that is the main contributor to the detector leakage current. Before the detector reaches full depletion, as the reverse bias voltage gradually increases, the depletion layer expands. Since the detector leakage current is proportional to the depletion depth, the leakage current increases linearly with the reverse bias voltage. When the reverse bias voltage reaches a certain threshold, the depletion layer extends to the entire detector thickness, at which point the detector reaches full depletion, the leakage current also reaches its maximum value (about 8 × 10^−7^ A). The magnitude of the leakage current directly affects the sensitivity and resolution of the detector. A larger leakage current increases the noise level of the detector, thereby reducing its signal-to-noise ratio. Therefore, controlling the leakage current is a crucial factor in improving detector performance.

**Figure 6:**
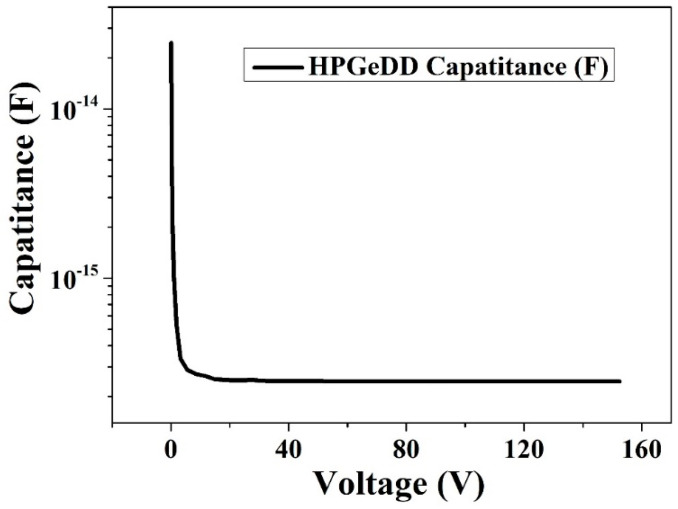
Capacitance versus voltage curve for the HPGeDD.

As shown in Figure 6, the graph illustrates the variation in capacitance with voltage for an HPGeDD. As the applied voltage gradually increases, the depletion region of the detector expands accordingly. Since the capacitance of the detector is inversely proportional to the thickness of the depletion depth, the capacitance of the detector gradually decreases as the depletion region expands. When the applied voltage reaches a full depletion voltage, the depletion depth is the detector thickness, and the capacitance no longer undergoes significant changes and stabilizes at 0.2 fF. The fully depleted state is the optimal state for detector performance because, in this state, all carriers within the entire volume can be efficiently collected, enabling highly effective signal response. The capacitance of the high-purity germanium drift detector proposed in this paper stabilizes at 0.2 fF in the fully depleted state, which is significantly smaller than the reported capacitance of 1 pF for point-contact electrode, high-purity germanium detectors [23], demonstrating a notable advantage of our detectors in capacitance characteristics.

## 5. Heavy Ion Incident Simulation

To investigate the carrier collection process of the HPGeDD under operating conditions, we conducted a simulation experiment involving heavy ion incidence on the detector. When the detector reached the fully depleted state, we selected a position at 750 μm along the *x*-axis as the incidence location, and a beam of heavy ions was vertically injected from the bottom of the detector. The initial time of incidence is at t = 5 ns, with an incidence depth of 200 μm. The radius of the interaction zone is set at 2 μm, and the linear energy transfer rate (LET) is set to 1 × 10^−5^ pC/μm.

**Figure 7:**
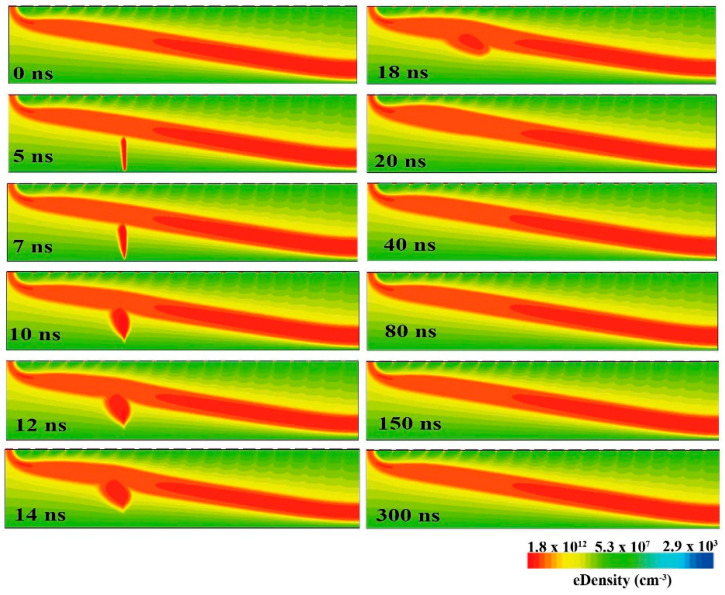
Simulation of heavy ion incidence on the HPGeDD. The incident time is 5 ns, and the incident position is x = 750 μm.

As shown in Figure 7, the image depicts the variation in hole density over time within the HPGeDD after the heavy ion incidence. Through a series of continuous images from 0 ns to 300 ns, we can clearly observe that when the heavy ions strike the detector, it deposits energy at the corresponding location. This energy deposition process subsequently excites the generation of electron–hole pairs within the sensitive area of the detector. Under the influence of the electric field, the holes move toward the hole drift channel and then toward the central collecting cathode, where they are eventually collected to form an electric signal. The continuous change in hole density over time in the simulation image showcases the entire process from the generation to the collection of induced holes. At 300 ns, the detector completes the charge collection and returns to its initial state. Throughout the entire process, from the moment the heavy ion incidence to the final completion of charge collection, the detector continuously generates a corresponding induced current signal.

**Figure 8:**
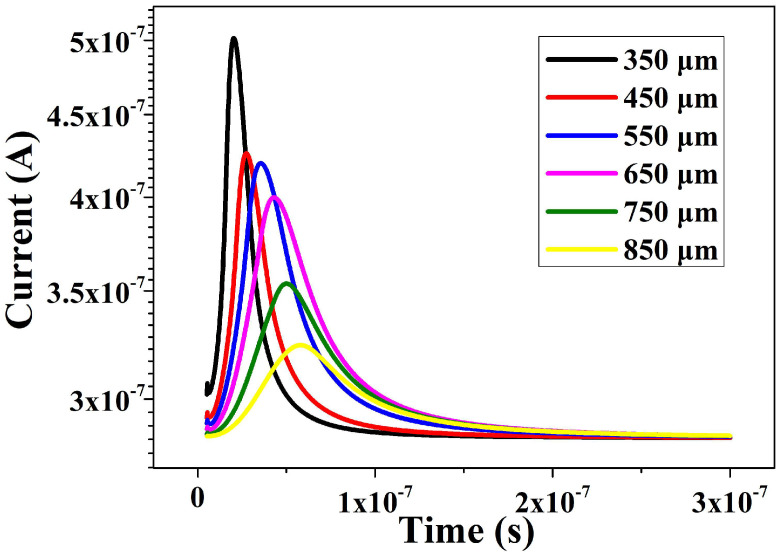
Curves showing the relationship between the current and time at the central cathode of the HPGeDD under heavy ion incidence at different positions.

As shown in Figure 8, the simulation of the induced currents by heavy ion incidence on the HPGeDD at different positions within the detector is presented, observing the current variation over time after the heavy ions enters the detector. For a single curve, at t = 5 ns, the moment of heavy ion incidence, the current value begins to appear and gradually increases. As the carriers are gradually transported to the central collecting cathode, the current value continues to rise until it reaches a peak. Subsequently, as a large number of carriers are collected by the central collecting cathode, the current gradually decreases to the lowest induced current level and eventually returns to the depleted state. By comparing different curves, it can be observed that positions closer to the central collecting cathode exhibit larger current peak signals, reach the peak earlier, and have the shortest signal collection time. This is because the distance for induced carriers to drift to the collecting cathode is shorter at positions closer to the cathode, resulting in a shorter collection time. Conversely, at positions farther away, the drift distance for induced carriers is longer, leading to a correspondingly longer collection time, a lower peak value, and a delayed time to reach the peak.

The simulation results above indicate that when using an HPGeDD, we should be mindful of the variations in signal response at different positions, with locations nearer to the central collecting cathode exhibiting higher signal amplitude and faster response times. This characteristic necessitates that we thoroughly consider the impact of positional effects on measurement outcomes when designing experiments or analyzing data. Furthermore, for measurement tasks requiring high-precision time resolution or strong signal peaks, it is advisable to prioritize detecting in the region close to the central collecting cathode to optimize detection efficiency and data quality.

## 6. Conclusions

Traditional HPGe detectors that aim to detect a broader range of radiation are often manufactured with centimeter-scale dimensions to achieve ultra-large sizes. However, the large size of HPGe detectors inevitably introduces the side effect of increased capacitance. Detector capacitance is a key factor affecting the noise level of the detector, and maintaining a low noise level is crucial for a stable detector performance. Reducing the overall size of the detector to decrease its capacitance would introduce a new issue with the reduction of the active area of the detector. On the other hand, the large volume of traditional HPGe detectors is not conducive to their application in aerospace and other fields where portability and small size are required.

To address this issue, this paper proposes an HPGeDD with a concentric ring structure based on the existing structure of drift detectors. The capacitance of this detector is only related to the area of the central collecting cathode, rather than the active area of the detector. Therefore, it is possible to significantly increase the overall area of the detector while keeping its capacitance low, thereby expanding the detection area of the detector. Electrical performance simulations of this detector structure were conducted using the semiconductor simulation software Sentaurus TCAD. The simulation results show that the potential distribution inside the detector is smooth, with no abrupt changes, and there is a lateral drift electric field parallel to the detector surfaces. The carrier drift channel pointing toward the central collecting cathode is clearly visible. Our detector has shown a low leakage current of 8 × 10^−7^ A and an extremely low capacitance of 0.2 fF. Furthermore, heavy ion incidence simulations were performed on the HPGeDD. The simulation results indicate that holes move along the carrier transport channel in the middle of the detector toward the central collecting cathode and are ultimately collected to generate an electrical signal. From the comparison of current signal curves, it can be observed that the closer the incidence position is to the center of the cathode, the shorter the collection time and the stronger the electrical signal.

To realize the HPGeDD proposed in this paper, key technologies that may be employed include atomic layer deposition (ALD) technology for ensuring uniform and pure deposition of Al_2_O_3_ on the wafer surface; ion implantation technology for precise control of doping concentrations; and microfabrication techniques such as photolithography and etching for fabricating the detector’s intricate structures. Additionally, packaging technology is required to protect the detector from external environmental interference, and a rigorous testing process is necessary to ensure stable and reliable detector performance, meeting the requirements for high-sensitivity and high-resolution detection.

Furthermore, we have outlined our next research steps, which involve conducting a systematic study on the radiation damage effects of HPGeDDs. This research aims to delve deeper into the mechanisms of radiation damage, exploring how external factors such as radiation dose and exposure time influence the performance of semiconductor detectors. Understanding these effects is crucial for the further development and optimization of HPGeDDs, and it represents an important extension of our current research efforts. Therefore, we plan to dedicate our upcoming work to this systematic study.

The research content of this paper is crucial for the subsequent development of HPGeDDs, as it can effectively optimize the performance of HPGe detectors and provide possibilities for their application in a wider range of fields.

## Figures and Tables

**Table 1:** The radian values correspond to resistor chains at different positions. The units for the radian values in the table are uniformly rad.

$\theta \left(r_{1}\right)=6.28$	$\theta \left(r_{2}\right)=3.86$	$\theta \left(r_{3}\right)=2.78$	$\theta \left(r_{4}\right)=2.18$
$\theta \left(r_{5}\right)=1.79$	$\theta \left(r_{6}\right)=1.52$	$\theta \left(r_{7}\right)=1.32$	$\theta \left(r_{8}\right)=1.16$
$\theta \left(r_{9}\right)=1.04$	$\theta \left(r_{10}\right)=0.94$	$\theta \left(r_{11}\right)=0.86$	$\theta \left(r_{12}\right)=0.79$
$\theta \left(r_{13}\right)=0.74$	$\theta \left(r_{14}\right)=0.69$	$\theta \left(r_{15}\right)=0.64$	$\theta \left(r_{16}\right)=0.60$
$\theta \left(r_{17}\right)=0.57$	$\theta \left(r_{18}\right)=0.54$	$\theta \left(r_{19}\right)=0.51$	

## Data Availability

The data presented in this study are available from the corresponding author upon reasonable request.
